# Visualizing the electron’s quantization with a ruler

**DOI:** 10.1038/s41598-021-89714-2

**Published:** 2021-05-25

**Authors:** Javier Tello Marmolejo, Mitzi Urquiza-González, Oscar Isaksson, Andreas Johansson, Ricardo Méndez-Fragoso, Dag Hanstorp

**Affiliations:** 1 Department of Physics, University of Gothenburg, 412 96 Gothenburg, Sweden; 2 Facultad de Ciencias, Universidad Nacional Autónoma de México, Av. Universidad 3000, Circuito Exterior S/N Delegación Coyoacán, C.P. 04510 Ciudad Universitaria, Ciudad de México, México

**Keywords:** Single photons and quantum effects, Optical manipulation and tweezers

## Abstract

More than 100 years ago, Robert Millikan demonstrated the quantization of the electron using charged, falling droplets, but the statistical analysis on many falling droplets did not allow a direct visualization of the quantization of charge. Instead of letting the droplets fall, we have used optical levitation to create a single droplet version of Millikan’s experiment where the effects of a single electron removal can be observed by the naked eye and measured with a ruler. As we added charges to the levitated droplet, we observed that its equilibrium position jumped vertically in quantized steps. The discrete nature of the droplet’s jumps is a direct consequence of the single-electron changes in the charge on the droplet, and therefore clearly demonstrates the quantization of charge. The steps were optically magnified onto a wall and filmed. We anticipate that the video of these *single electron additions* can become a straightforward demonstration of the quantization of charge for a general audience.

The idea of quantization is one of the most important concepts in physics and an essential component of our conceptualization of the microscopic world. Nevertheless, because of the very fact that it concerns the microscopic world, macroscopic observations of quantization are rare.

Since their invention in 1970 by Nobel Prize winner Arthur Ashkin, optical trapping techniques have allowed us to isolate and manipulate micro- and nano-particles^1^. This can now be performed so precisely that relatively large objects can be employed to explore quantum phenomena. Examples include the search of milli-charged particles^2^, collisions of ultracold ground-state molecules^3^, and the investigation of macroscopic quantum states^4^. These experiments, along with others like the visualization of quantized vortices^5^ or of quantum cat states^6^, continue to bring the concept of quantization to the macroscopic world.

In this paper, we add a new macroscopic visualization of quantization through a modern single-droplet version of Millikan’s experiment^7^. In past experiments, quantized amounts of electric charge in the form of electrons have been added to trapped particles using optical^8–10^ and electrostatic^11^ levitation. However, these observations were not direct and necessitated the use of feedback loops or post-experiment mathematical analysis to discern the quantization. The technique we present here is direct and shows the magnified effect of adding individual electrons to trapped particles live on a screen and visible with the naked eye.

The quantization was observed in the stability position of a $29.5 \pm 1.4\,\upmu$m silicone oil droplet optically levitated between two horizontal electrodes. A potential difference between the electrodes produced a vertically-directed and locally homogeneous electric field around the droplet. The net amount of charge on the droplet created a force that displaced it in the vertical direction. Since the optical trap held the droplet in a harmonic potential, the displacement was proportional to the force, which, in turn, depended on the droplet’s net amount of charge. The position dependence of the droplet’s net charge was sufficiently enhanced to clearly observe the result of depositing individual electrons on the levitated droplet.

The technique we used to visualize the effect of adding individual electrons to the droplet requires three elements: (i) an optical levitation trap with a very low trap stiffness (i.e. the spring constant of the harmonic potential) to make the movement per unit force large, (ii) a strong electric field, and (iii) a method of adding individual electrons to the levitated droplet.

We created the trap by directing a 532 nm continuous wave laser beam (LaserQuantum gem532) vertically upwards and focusing it with a 100 mm plano-convex lens. The long focal distance produced a weak optical trap. The laser beam had a diameter of 0.9 mm, resulting in a numerical aperture (NA) of $4.5 \times 10^{-3}$ and in a trap stiffness of $5.00 \pm 0.49$ nN/m (see Suplementary Information).

We produced the electric field by applying a voltage difference of 666 $\pm 0.5$ V across a pair of horizontal electrodes with a vertical separation of 1.0 mm. The laser passes through each electrode via a circular hole with a radius of 1.0 mm. The small distance between the electrodes and the large voltage difference were chosen to produce a strong electric field.

We used a numerical simulation to determine the magnitude and homogeneity of the electric field. We defined a working volume between the electrodes inside of which we know the experiment took place. In this volume, the electric field was found to have a vertical direction and a magnitude of $360 \pm 45$ kV/m. In a smaller volume sufficient for the droplet to make a couple of jumps, the homogeneity of the field is even greater, which results in equispaced jumps (see Supplementary Information).

We added individual electrons to a previously neutralized droplet (see Supplementary Information) using a ${}^{241}\text {Am}$ alpha radiation source. The emitted alpha particles produced free electrons in the trap either directly by striking the droplet or indirectly by striking the electrodes or ionizing the air in the trap. The free electrons were subsequently deposited on the surface of the droplet. In this manner, we were able to change the charge on the droplet in randomly distributed steps of either single or small multiples of the elementary charge.

**Figure 1:**
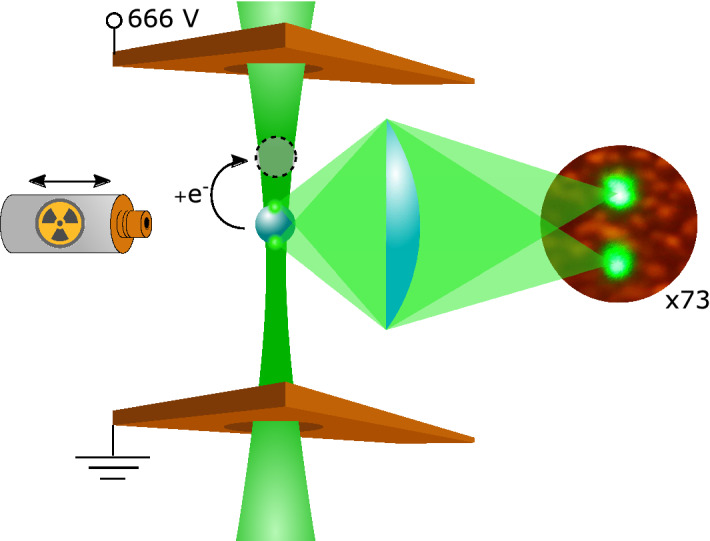
Schematic of the experimental setup. A silicone oil droplet is levitated between two flat, horizontal electrodes with a separation of 1.0 mm and a voltage difference of 666 $\pm 0.5$ V. A ${}^{241}\text {Am}$ alpha radiation source is placed close to the droplet in order to create free electrons. Some of these electrons become attached to the droplet and change its net charge. The levitated droplet scatters mostly from the bottom and top where the laser enters and exits the droplet. This creates a double point image when focused by a lens.

We projected an image of the droplet onto a wall in the laboratory using an aspheric planoconvex lens (f = 50 mm, Thorlabs AL2550G) with a magnification of $73 \pm 1.4$. With this magnification, we were able to observe micro-metric movements of the droplet with the naked eye. The light scattered from the droplet comes mostly from the bottom where the laser beam hits the droplet and the top where the beam leaves the droplet. Hence, the image of the droplet is observed as two separate images on the screen, as seen in Fig. 1.

When we set the alpha radiation source at the appropriate distance from the droplet, it gains charges randomly. This causes it to jump discontinuously from one equilibrium point to another. We filmed a video of a series of electron additions (see online). Some selected frames are shown in Fig. 2a. Between each of the frames, the droplet gained an additional amount of charge causing it to jump from one equilibrium position to another. Equally spaced horizontal reference lines are added to Fig. 2a. The separation between the equally spaced horizontal lines corresponds to the distance the droplet moves when absorbing a single elementary charge. The quantization of the charge immediately stands out. The position of the droplet always falls on one of the horizontal lines and, by simply counting the number of lines between the steps, one can determine the number of electrons the droplet has gained.

**Figure 2:**
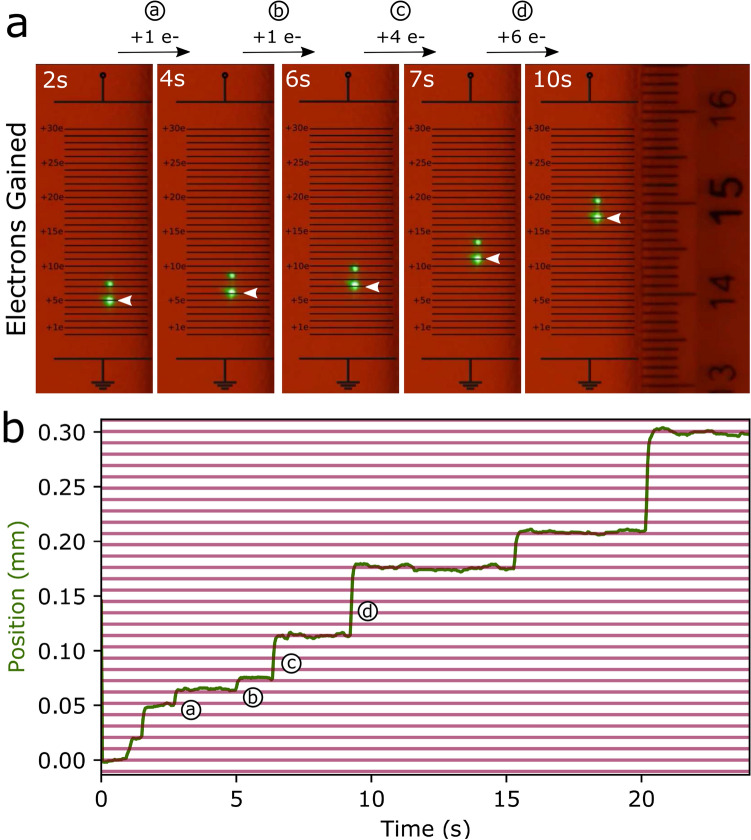
Visibly quantized steps of a levitated droplet as it gains electrons. (**a**) Screenshots of the droplet’s projection on the wall before and after steps a (+ 1e), b (+ 1e), c (+ 4e) and d (+ 6e). Black horizontal lines mark the displacement caused by a single electron addition and are set to the lower bright image of the droplet. The last image to the right shows a mm ruler that had been attached to the wall. (**b**) Real displacement of the droplet (green) calculated from the video of the displacement on the wall.

**Figure 3:**
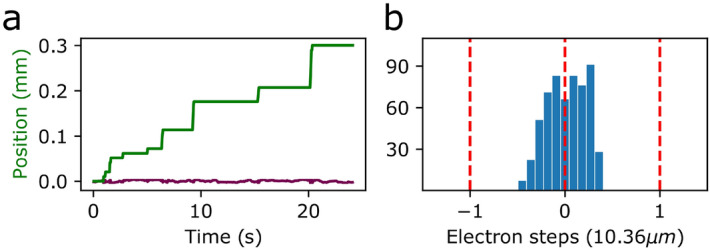
Discrete steps in the vertical position of droplet in the trap. (**a**) The green line shows a fit of the data shown in Fig. 2b using a step function. The height of the steps is an integer multiple of the *single electron step* fitting parameter (see discussion in text). The magenta line shows a plot of the residue between the data and the fit. (**b**) Histogram of the residue measured in units of electron steps. The normal distribution around zero results from the discrete nature of the steps.

Figure 2b presents the position as function of time for the droplet shown in the video. The magenta equally spaced lines represent the displacement, $\Delta y$, caused by a single electron addition and all the steps are multiples of this distance. In this graph, one can clearly see the full series of 8 steps that fall on a horizontal line, where the droplet gains 2, 3, 1, 1, 4, 6, 3, and 9 electrons. Once again, one can observe the quantization of the electronic charge arising from the discrete nature of the individual steps.

We determined the displacement $\Delta y$ by fitting the positions of the droplet to a step function, which is shown in Fig. 3a. The fit produced a value of $\Delta y$ of $10.36 \pm 0.26 ~\upmu$m, which is almost three times smaller than the droplet’s diameter. The two fit variables were a global *single electron displacement* $\Delta y$, and an individual *number of electrons added* for each point in time. The uncertainty in $\Delta y$ stems mostly from the droplet’s oscillation around the stability positions.

The residue between the fit and the data is plotted in magenta at the bottom of Fig. 3a and a histogram of this residue is plotted in Fig. 3b. The histogram follows a normal distribution around zero and its FWHM is less than half an electron step, providing further evidence that we are observing quantized steps.

To confirm that these steps were indeed caused by electron additions, we used them to calculate the charge of the electron. Balancing the electrostatic ($F_e = q E$) and optical restoring ($F_r = k \Delta y$) forces results in

1 $$\begin{aligned} q = \frac{k \Delta y}{E}, \end{aligned}$$

where *q* is the charge of the electron, $\Delta y$ the displacement caused by a single electron addition, *k* the trap stiffness and *E* the magnitude of the electric field. The method to determine the trap stiffness is described in the methods section. Using Eq. (1), we calculated the charge of the electron to be of $1.44 \pm 0.25 \times 10^{-19} C$, which agrees within the statistical uncertainty with the known value of $1.602 \times 10^{-19} C$^12^. The uncertainty was calculated through error propagation in Eq. (1) where the biggest contributors were the uncertainties of the electric field and the trap stiffness.

The series of electron jumps serve as straight forward evidence of the quantization of the electric charge. We have magnified the effect to a level where the step caused by adding a single electron can be seen with the naked eye and measured with a simple ruler. The discrete steps are the result of the charge on the droplet changing by a single electron. In contrast, other methods of observing the effects of quantization such as the photoelectric effect or atomic emission lines are indirect in the sense that they involve the use of many photons or electrons. Our experiment allows one to directly visualize charge quantization, a quantum phenomenon, in the macroscopic world.

## Supplementary Information

Supplementary Information.

Supplementary Video.

## Data Availability

All relevant data generated or analysed for this study are available within the article and the associated Supplementary Information. Any other data are are available from J.T.M (javier.marmolejo@physics.gu.se) upon reasonable request.
