# Supplementary material for: Visualizing the electron’s quantization with a ruler
Source: Sci Rep. 2021 May 25;11:10703. doi: 10.1038/s41598-021-89714-2 (PMC8149853; doi:10.1038/s41598-021-89714-2)
Supplement: Supplementary file 1 — Supplementary Information. [file 41598_2021_89714_MOESM1_ESM.pdf]

Supplementary Information

Visualizing the electron's quantization with a ruler

Javier Tello Marmolejo,[1,*] Mitzi Urquiza-González,[1,2] Oscar Isaksson,[1] Andreas Johansson,[1] Ricardo Méndez-Fragoso[2] and Dag Hanstorp[1]

[1]Department of Physics, University of Gothenburg, SE-412 96 Gothenburg, Sweden

[2]Facultad de Ciencias, Universidad Nacional Autónoma de México, Av. Universidad 3000, Circuito Exterior S/N Delegación Coyoacán, C.P. 04510 Ciudad Universitaria, Ciudad de México, México

[*]javier.marmolejo@physics.gu.se

Supplementary Video

This video shows a projection of the levitated droplet on a screen. As the droplets gains electrons it jumps quantized amounts. Horizontal lines are superimposed to mark the distance the droplet jumps when it absorbs one electron. The lines are adjusted to the lower bright spot.

Conceptual Image

The main result of this paper can be instantly understood from the sketch shown in Fig. 1 where small amounts of electrons are consecutively added to the levitated droplet. Here it is shown how the size of the steps between each equilibrium position is quantized and can be measured with a simple ruler. This is a conceptual image that shows the stability position of the droplet at different times as shown in the Supplementary Video.

Calculation of the trap stiffness

The left inset in Fig. 2 shows the movement of the droplet during a period of 1.2 seconds recorded with a position sensitive detector placed on the image plane projected by the lens. A short and very stable period is chosen to ensure that the movement is solely caused by thermal noise. A histogram of this data is shown in the inset to the right. If one assumes the droplet resides in a harmonic potential provided by the trap, the histogram represents the probability distribution of the droplet. Using Boltzmann statistics, the logarithm of the

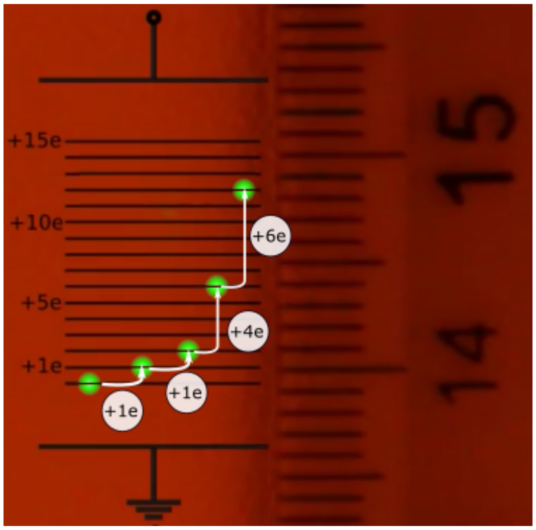

FIG. 1. Quantized jumps of an optically trapped droplet inside a strong, vertical electric field as electrons are added. Conceptual image adapted from data where 10.36 ± 0.26 μm steps are magnified 73 ± 1.4 times and shown on the wall in the laboratory. A simple ruler shown to the right was included to establish a length scale.

histogram multiplied by $-k_B T$ results in the potential felt by the droplet. The potential was fitted with a parabola to obtain the trap stiffness. More details can be found in a similar calculation performed by Florin et al.[1]

Droplet Neutralization

We found that dispensed droplets have some random amount of charge of around a few hundreds of elementary charges. We measured this by applying a weak AC field that made the droplet oscillate up and down. The amplitude of the oscillation depended on the magnitude of the charge on the droplet. We then moved the alpha radiation source closer to the droplet to change its charge. This allowed us to observe the effect of the oscillatory electric field on the droplet with net negative, neutral and positive charges. The amplitude of the oscillations of the droplet depended on the magnitude of its net charge. The response is shown in Fig. 3. At t=9 s, marked with a vertical line, the oscillations of the droplet (green) are out of phase with the applied AC field (magenta). This corresponds to a net positive charge on the droplet. Later, at the second vertical line at t= 61 s, the two oscillations are in phase, which corresponds to the droplet having a net negative charge. In between these limits, in the region around t=40 s, there are no oscillations observed since the droplet is

almost electrically neutral. For the experiments shown above, the source was removed when the minimum amplitude was met. This process was repeated with increasing AC voltages until the charge of the droplet was so low that the 666 V DC could be applied without the droplet moving out of the working volume. At this stage, the droplet was prepared to conduct the experiment explained in the main body of the paper.

Simulation and measurement of the electric field

The magnitude and direction of the applied electric field were determined using a numerical simulation that took into account the geometry of the plates. Fig. 4**A** is a schematic diagram showing the upper and lower plates. A voltage is applied across the plates with the bottom plate being grounded. The path of the laser beam is shown in green and the dotted square indicates the working volume where the experiment was performed. We defined this working volume as a cube with 0.8 mm sides in the center of the electrodes. This region represents the volume that encompasses the droplet since the electrodes are radially symmetric around their apertures. The simulation was performed using the Gauss – Seidel method on a mesh that takes into account the rounded edges of the electrodes. The simulation showed that

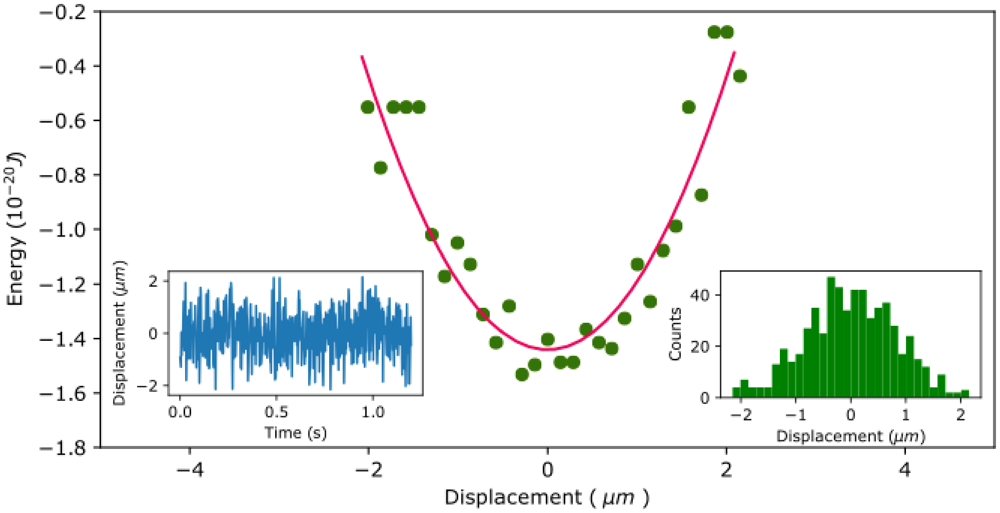

FIG. 2. Harmonic potential (green dots) felt by the droplet fitted with a parabola (magenta line) to determine the trap stiffness. The potential was determined using the histogram (right inset) which displays the scatter in the position of the droplet due to thermal fluctuations over a period of 1.2 seconds (left inset). The logarithm of the histogram times the Boltzmann factor, $-k_BT$, results in the potential.

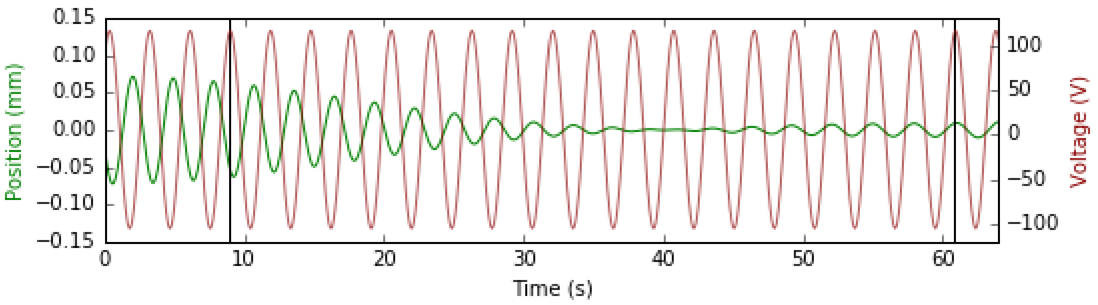

FIG. 3. An optically levitated droplet is irradiated by alpha particles from a radioactive source, resulting in it accumulating electric charge on its surface. An applied AC electric field (magenta) forces the droplet to oscillate (green) with an amplitude that depends on the magnitude of its charge. It can be seen that at t= 9 s the net charge is positive and the oscillations of the droplet and applied electric field are out of phase. At t=61 s, the droplet has a net negative charge and it oscillated in phase with the electric field. In between, at t=40 s, the droplet is essentially electrically neutral and its oscillation has been damped.

between any two points separated by 50 μm inside the working volume, the electric field changed by a minimum of 0.15% close to the center and a maximum of 5.1% close to the edges.

Fig. 4**B** shows the trajectories of particles inside the calculated electric field that follow electric field lines. It can be seen that the dominant component of the electric field is in the vertical direction. Thus we conclude that the electric field within the working volume is locally homogeneous.

---

[1] Florin, E.-L., Pralle, A., Stelzer, E.H.K. & Hörber, J.K.H. Photonic force microscope calibration by thermal noise analysis. Appl. Phys. A **66** 75—78 (1998).

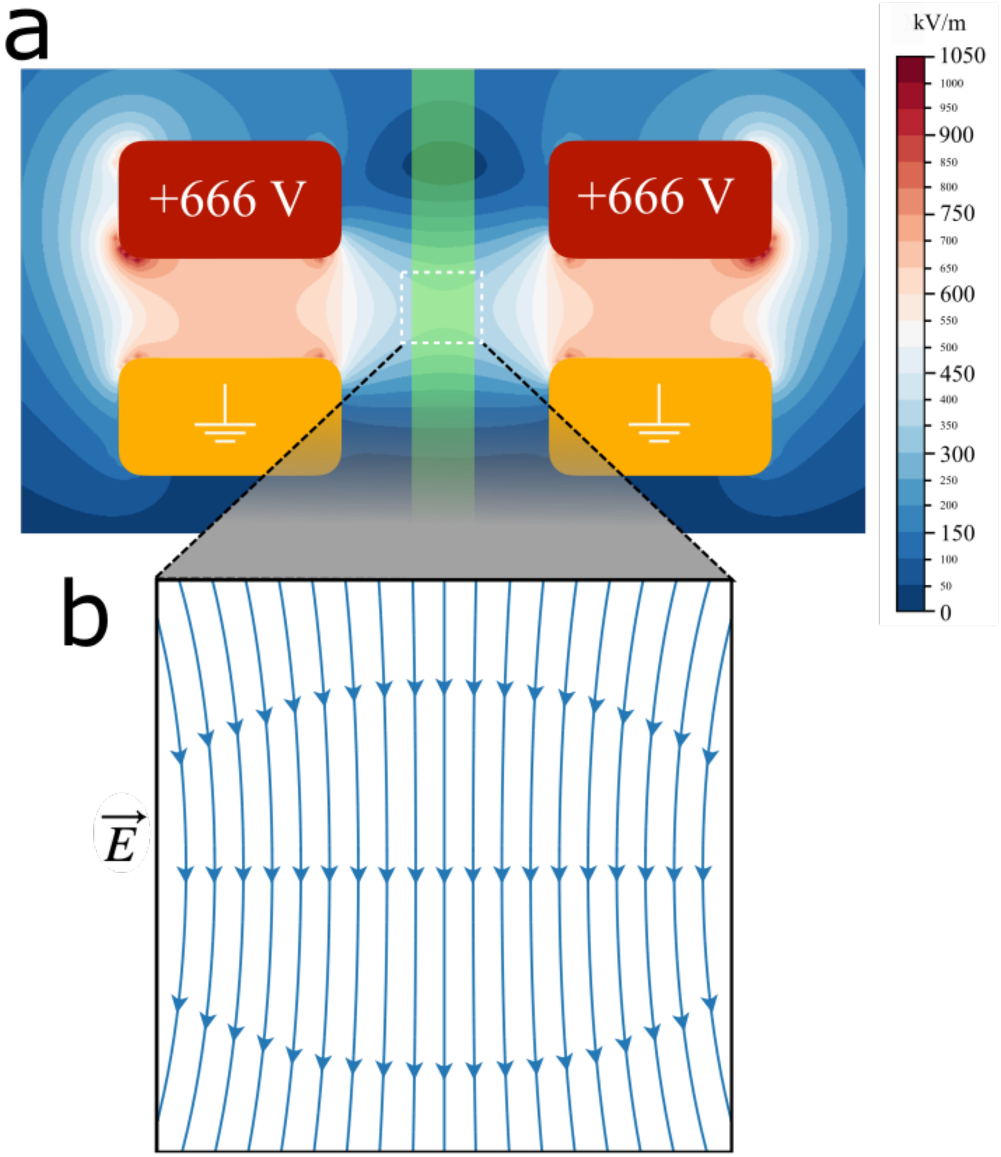

FIG. 4. The result of a numerical simulation of the expected magnitude and direction of the electric field produced by electrodes. The figure demonstrates local homogeneity in a volume surrounding the trapped droplet. **A** The colors show the magnitude of the electric field. The working volume is denoted by the dotted square, inside of which the magnitude of the electric field was found to be 360 ± 45 kV/m. **B** Plot of the electric field lines calculated by following the trajectory of initially static, positively charged particles. The starting points of the trajectories were chosen uniformly distributed along the horizontal middle zone of the work volume. The numerical simulation shows that the electric field is essentially vertical inside the working volume.
